# Generalized Anxiety Disorder, Depressive Symptoms, and Sleep Problem During COVID-19 Outbreak in Ethiopia Among Police Officers: A Cross-Sectional Survey

**DOI:** 10.3389/fpsyg.2021.713954

**Published:** 2021-09-09

**Authors:** Mekonnen Tsehay, Moges Necho, Habtam Gelaye, Abeba Beyene, Mengesha Birkie

**Affiliations:** Department of Psychiatry, College of Medicine and Health Science, Wollo University, Dessie, Ethiopia

**Keywords:** COVID-19, depression, general anxiety, sleep quality, coping status

## Abstract

**Background:** Coronavirus disease 2019 (COVID-19) is an outbreak that caused serious threats to people worldwide. Police officers are one of those frontline fighters during pandemic. Our study is the first to examine psychological health response among police officers in Ethiopia during the COVID-19 outbreak.

**Methods:** A cross-sectional study design with a self-administered questionnaire was conducted among police officers from Dessie town from June 20 to July 10, 2020. A total of 385 questionnaires were completed correctly accounting for 91% of the total. The data were collected by using demographic information and psychological health assessment tools. The Patient Health Questionnaire (PHQ-9), Generalized Anxiety Disorder 7-item scale (GAD-7), Insomnia Severity Index (ISI), and Brief Resilient Coping Scale questionnaire were used to assess depression, anxiety, sleep, and coping status of participants.

**Results:** The rate of depression was found to be 28.9%. Of these, 19.7% had mild, 7.3% had moderate, 1.6 had moderate–severe, and 0.3% had severe depression symptoms. The rate of general anxiety symptoms was found to be 30.2%. Of these, 22.1% of the police officers had mild, 2.6% had moderate, and 5.5% had severe anxiety. Moreover, 13.8% of police officers had subthreshold insomnia and 2.1% had clinical (moderate–severe) insomnia. Participants who are men, married, highly resilient, and have high social support were associated with lower depression, anxiety, and insomnia scores than those of women, being single or widowed/divorced, low resilient coping score, and low social support, respectively.

**Conclusion:** A psychological health problem was found to be higher among police officers in Dessie town. Younger age, sex, marital status, having chronic diseases, coping, and social support with depression, general anxiety, and insomnia were found to be significantly associated with psychological health problems. There is a need for mental health services, support, and care of police officers during the pandemic.

## Background

Coronavirus disease 2019 (COVID-19) is an outbreak; even if it started in China, now it is a threat illness worldwide, including Ethiopia (Malik et al., 2020). Onset of sign and symptoms may be started within 2 days or as long as 2 weeks after being infected. Fever, cough, difficulty in breathing, and shortness of breath are the most common symptoms that are reported by patients (Ganyani et al., 2020; Huang et al., 2020).

A virus causing COVID-19 is named as severe acute respiratory syndrome coronavirus-2 (SARS-CoV-2) (Lai et al., 2020). Respiratory droplets were thought to be the main mechanism of transmission from person to person among close contacts. These droplets are produced when an infected person coughs, sneezes, or talks and can land in the mouth or nose, or may be inhaled into the lungs, of people who are nearby (Shereen et al., 2020).

The COVID-19 virus has spread across the global exploding into a world pandemic for several reasons: it is transmitted by asymptomatic patients, it is highly contagious, and a few infected individuals do not experience any symptoms (Cucinotta and Vanelli, 2020).

At present, increased global mobility has provoked new outbreaks. The increasing number of patients and suspected cases has caused the general public to become infected. Additionally to healthcare workers, law enforcement officials are bravely fighting on the front lines of the pandemic (Stogner et al., 2020).

Policing is one in every of the foremost mentally challenging occupations, coping with long and infrequently rotating shifts, threats of violence, increased need for hypervigilance, and an absence of public support creating chronic stress (Carlier et al., 1997; McCraty and Atkinson, 2012).

Police services or military units charged with civil policing need to enforce and monitor these restrictions. These frontline fighters not only face an identical risk of infection because of the general public but also suffer from fatigue caused by overtime working and as well as the pressure of responsibility (Cai et al., 2020; Sun et al., 2020). In case of developing countries such as Ethiopia, these frontline fighters work without sufficient self-protective equipment and low access to healthcare services if infected, which increases the burden significantly compared to the developed countries. It results in a large number of psychological problems, such as anxiety, depression, and sleep problems, among police officers (Du et al., 2020; Stogner et al., 2020).

The aim of this study was to determine the prevalence and severity of psychological health problems among law enforcement officials during the COVID-19 pandemic, and thus it will give basis for implementing relevant psychological state intervention measures to cope with the challenge effectively. A self-administered questionnaire was used to investigate anxiety, depression, sleep status, and coping among law enforcement officials including criminal police, security police, special police, traffic police, the police of logistics support, and prison guards in Dessie town, who were in duty at the time of information collection.

## Materials and Methods

This study utilized a cross-sectional survey design to examine the prevalence and factors associated with anxiety, depression, and sleep problems in frontline law enforcement officials.

### Study Setting

Data were collected from June 20 to July 10, 2020 in Dessie town during the COVID-19 pandemic. Consent was provided by the subjects before study commencement. Subsequently, we distributed self-report questionnaires to the police officers who were in duty.

### Sampling Procedures

Convenience sampling method was used because we cannot find the precise number of police officers in Dessie town, and that we tried to incorporate from all style of cops (criminal police, security police, special police, traffic police, police of logistics support, and prison guards) who were on duty at the time of information collection. In addition to ethical approval obtained from Institutional Review Board (IRB) of Wollo University and permission obtained from administrative bodies in Dessie town police organization, written informed consent was obtained from each participant. The right of respondents not to give information about their privacy was reassured and any information obtained were kept confidentially. During data collection, the purpose of the study was properly clarified to the respondents and the questions were delivered in their own language. Those who scored severe psychological problems (e.g., depression, anxiety, and insomnia) were advised to contact the mental health specialist (principal investigator).

### Sample Size Determination

Single population proportion formula was used to determine the sample size by considering that 95% of confidence interval, proportion of 50, and marginal error (d) of 5% were accustomed to maximize sample size. Also, 10% was used for non-response rate, and the ultimate sample size was 384 + 39 = 423.

### Study Participants

As a study participant, we included all police officers aged above 18 years, whether or not they were criminal police, security police, special police, traffic police, police of logistics support, and prison guards, who were on duty at the time of knowledge collection in Dessie town.

### Materials

Questionnaires were developed to assess the demographic characteristics such as gender, age, legal status, level of education, hospital department, and city.

To screen depression, we used the Patient Health Questionnaire (PHQ-9). It is a 9-point item questionnaire that is scored from 0 to 3 generating a complete score starting from 0 to 27. A complete score of 0–4 indicates minimal depression, 5–9 mild depression, 10–14 moderate depression, and 15–19 moderately severe depression, and 20–27 severe depression. The specificity (67%) and sensitivity (86%) of the questionnaire was validated in Ethiopia with Amharic version with Cronbach’s alpha of 0.85 (Gelaye et al., 2013).

To assess the general anxiety, generalized anxiety disorder-7 (GAD-7) was used and it is a 7-point item questionnaire and every item incorporates a 4-point Likert scale that ranges from 0 to 3, where a complete score ranges from 0 to 21. The intervals 5–9, 10–14, and 15–21 represent cut points for mild, moderate, and severe anxiety, respectively. It is designed primarily as a screening and severity measure for GAD, and it is also a good tool for screening with cut point score of 10 or greater (Spitzer et al., 2006).

#### Insomnia Severity Index

It is a 7-item questionnaire with a 5-point Likert scale ranging from 0 to 4 where for items 1–3, 0 means “none” and 4 means “very severe,” for item 4, 0 means “satisfied” and 4 means “very dissatisfied,” for item 5, 0 means “not in any respect noticeable” and 4 means “very much noticeable,” for item 6, 0 means “not in any respect worried” and 4 means “very much worried,” and for item 7, 0 means “not the least bit interfering” and 4 means “very much interfering”. However, the severity of insomnia cannot be measured. A complete score ranges from 0 to 28. Variants 0–7 = no clinically significant insomnia; 8–14 = subthreshold insomnia; 15–21 = clinical insomnia (moderate severity); and 22–28 = clinical insomnia (severe) (Manzar et al., 2020).

#### Brief Resilient Coping Scale

A 4-item questionnaire with a 5-point Likert scale ranges from 1 to 5, where 1 means “not describe me at all” and 5 means “describes me very well” and its accustomed screen coping mechanisms. Various 4–13 points are low resilient copers, 14–16 points = medium resilient copers, and 17–20 points = high resilient copers (Ahern et al., 2006).

#### Oslo Social Support Scale

Oslo Social Support Scale (Oslo-3) wont to screen the provision of social support. It is a 3-item questionnaire that ranges from scores 3 to 8 = poor support, 9 to 11 = moderate support, and 12 to 14 = strong support (Kocalevent et al., 2018).

### Statistical Data Analyses

Continuous and categorical variables were summarized as mean values ± variance (SD) and frequency (percentage), respectively. Assumptions were checked first and then univariate and multivariate regressions toward the mean analysis model was fitted to spot the connection between sociodemographic factors and dependent variables (i.e., depression, anxiety, and sleep problems). Statistically significant differences were identified as a two-sided *p*-value < 0.05.

## Results

### Study Participants Demographic Data

Data from a complete set of 385 eligible participants were included within the end for a participation rate of 91% (423 of 385 participants). Of the entire sample, 385 participants (84.2%) were men, and therefore the mean (SD) age was 34 (7.42) years; during this study, all law enforcement officials reported as they or their members of the family did not have any history of being quarantined or being infected with COVID-19. Of the full number of respondents, 197 (51.2) live alone, 156 (40.5) support members of the family, and 32 (8.3) board apartments. The bulk of law enforcement officials are single, 266 (69.1%) and 14 (3.6%) are widowed/divorced. From all the participants, 19 (4.94%) have a history of chronic medical illness, either hypertension, diabetic mellitus, cardiac illness, or asthmatic illness (Table 1).

**TABLE 1:** Socio-demographic data participants and binary linear regression analysis of depression, anxiety, and sleep problem.

Socio-demographic variables			Binary regression					
variables	Variable category	*N* (%) or μ (SD)	Depression		Anxiety		Sleep	
			B	Sig.	B	Sig.	B	Sig.
Age		34 (7.42)	0.154	0.003	0.015	0.630	0.095	0.001
sex	Male	324 (84.2)	–0.497	0.04	–1.965	0.002	–0.148	0.800
	Female	61 (15.8)	0		0		0	
Marital status	Married	105 (27.3)	0.424	0.353	0.262	0.004	0.1528	0.001
	Single	266 (69.1)	0.048	0.920	–0.567	0.278	1.736	0.001
	Divorced/widowed	14 (3.6)	2.314	0.039	1.613	0.195	–0.513	0.652
Religion	Orthodox	199 (51.7)	0.190	0.653	–0.603	0.195	0.079	0.853
	Muslim	149 (38.7)	–0.123	0.776	0.060	0.901	–0.400	0.361
	Protestant	21 (5.5)	0.470	0.613	3.886	0.001	2.171	0.020
	Others	16 (4.2)	–1.064	0.314	–0.893	0.444	–0.926	0.386
Current living with	Alone	197 (51.2)	–0.137	0.745	–0.380	0.415	0.662	0.144
	With family	156 (40.5)	0.182	0.672	0.786	0.097	–0.641	0.140
	In apartment	32 (8.3)	–0.124	0.821	–1.241	0.141	–0.014	0.986
Having chronic illness		19 (4.94)	0.934	0.037	0.532	0.021	0.520	0.098
BR Coping score		14.46 (4.23)	–0.132	0.008	–0.174	0.02	–0.093	0.066
Social support		7.94 (3.67)	–0.109	0.008	–0.104	0.025	0.180	0.025

### Prevalence of Symptoms of Depression, Anxiety, and Sleep Quality

For reliability of scales, we found alpha value = 79.7, 83.2, and 91.4% for PHQ-9, GAD-7, and Insomnia Severity Index (ISI) which shows very good and above level in our study participants.

The prevalence of symptoms for psychological problems among the full sample was 28.9% (95% CI, 26.5–30.2%) have depression, with 19.7% mild depression, 7.3% moderate depression, 1.6% moderate–severe depression, and 0.3% severe depression. 30.2% (95% CI, 28.2–33.0%) have anxiety with 22.1% mild anxiety, 2.6% moderate anxiety, and 5.5% severe anxiety. 15.9% (95% CI, 13.5–17.2%) for insomnia. The majority of participants have good knowledge of COVID-19 transmission and pandemic (Table 1).

### Factors Associated With Symptoms of Depression, Anxiety, and Sleep Quality

In the multivariable analysis, being younger age, sex, marital status, having chronic diseases, coping, and social support were still found to be associated with the symptoms of depression, anxiety, and insomnia. Male participants and coping score people displayed a remarkably higher risk for depression, anxiety, and marital status for insomnia (Table 2).

**TABLE 2:** Prevalence report of participants on knowledge about COVID-19, depression, general anxiety, coping status, and sleep quality.

Variable	category	frequency	Percentage
Knowledge of covid-19 transmission	By Droplet	383	99.5
	By breathing	380	98.7
	By material	385	100
COVID-19 is pandemic (worldwide disease)		385	100
History of quarantine or being infected with COVID-19/you or your family member?		0	0
Depression	Minor/no depression	274	71.2
	Mild depression	76	19.7
	Moderate depression	28	7.3
	Moderate severe	6	1.6
	Severe depression	1	0.3
Coping	Low resilient copers	124	32.2
	Medium resilient copers	117	30.4
	High resilient copers	144	37.4
General Anxiety	No anxiety	269	69.9
	Mild anxiety	85	22.1
	Moderate anxiety	10	2.6
	Severe anxiety	21	5.5
Sleep problem	No clinical insomnia	324	84.2
	Sub-threshold insomnia	53	13.8
	Clinical insomnia (moderate severe)	8	2.1
History of quarantine or being infected with COVID-19		0	0

Male individuals showed 0.312, 0.865, and 0.035 times reduction in depressive, anxiety, and insomnia symptoms, respectively, when compared with those female participants (B, -0.312, *p*-value = 0.001) for depression, (B, 0.865, *p*-value = 0.004) for anxiety, and (B, 0.035, *p*-value = 0.05) for insomnia. Age of the participants was also a significant predictor of depression and insomnia symptoms with (B, 0.132, *p*-value = 0.002) and (B, 0.135, *p*-value = 0.001), respectively. In addition, associations were identified between marital status and three psychological health problems, namely, depression, anxiety, and insomnia. Being divorced/widowed increases depression symptoms (B, 1.256, *p*-value = 0.002), and being single decreases anxiety symptoms (B, 0.213, *p*-value = 0.008) but increases insomnia symptoms (B, 0.892, *p*-value = 0.001).

Having one or more chronic medical illnesses, from hypertension, diabetic mellitus, and cardiac illness, to asthma increases all the three psychological health problems (B, 1.235, *p*-value = 0.001 for depression, B, 0.825, *p*-value = 0.004 for anxiety, and B, 0.321, *p*-value = 0.002 for insomnia).

However, social support and resilient coping score reduced the score of psychological health problems, namely, depression, anxiety, and insomnia as shown in Table 3.

**TABLE 3:** Multivariate analyses of factors related to depression, anxiety, and insomnia.

Socio-demographic variable		Multivariate linear regression					
Variables	Variable category	Depression		General Anxiety		Sleep quality	
		B	Sig.	B	Sig.	B	Sig.
Age		0.132	0.002	0.115	0.070	0.135	0.001
Sex	Male	–0.312	0.001	–0.865	0.004	–0.035	0.05
	Female	0		0		0	
Marital status	Married	0		0		0	
	Single	0.124	0.521	–0.213	0.008	0.892	0.001
	Divorced/widowed	1.256	0.002	0.219	0.321	–0.621	0.120
Having chronic illness		1.235	0.001	0.825	0.004	0.321	0.002
BR Coping score		–0.521	0.001	–0.541	0.002	–0.393	0.021
Social support		–0.032	0.001	–0.201	0.005	0.236	0.054

## Discussion

We found that psychological health problems among police officers in our study area were high, which shows it as a public health concern. The prevalence of symptoms for psychological problems among the full sample was 28.9% (95% CI, 26.5–30.2%) have depression, 30.2% (95% CI, 28.2–33.0%) have anxiety, and 15.9% (95% CI, 13.5–17.2%) for insomnia.

The prevalence of psychological health problems found in the current study was similar with a prevalence study that was conducted among the overall population in China, which indicated that just about 34.4% of the respondents manifested depressive symptoms during the COVID-19 outbreak (Kang et al., 2020). Almost similar findings reported by another study done by Liang et al. (2020) in China showed that 30.43, 20.29, and 14.49% of frontline fitter doctors have health problems, such as depression, anxiety, and insomnia, during the COVID-19 pandemic.

A survey done in late January 2020 found that about one-third of respondents experienced moderate-to-severe psychological health problems which is higher than pre-outbreak prevalence report (Ahmed et al., 2020).

The results obtained *via* another online survey of Chinese adolescents revealed that the prevalence rates of health symptoms such as depression, anxiety, and a mix of depression and anxiety were 43.7, 37.4, and 31.3%, respectively (Zhou et al., 2020). In a study carried out in Singapore, the prevalence rate was reported as 22.9% (Sim et al., 2010). The difference can be due to the population and the tools they used. In China, the adolescents were included by online data collection, and in Singapore, general health questionnaires folks who came for a clinical visit were included.

A report among frontline working doctors from Bangladesh also shows slightly higher prevalence rates of anxiety, depression, and insomnia, i.e., 36.5, 38.4, and 18.6%, respectively (Barua et al., 2020). The possible reason might be the differences in population and the tools they used, e.g., Patient Health Questionnaire-4 and two-item version of the Sleep Condition Indicator.

The prevalence of symptoms of depression was beyond the previous National Health Survey in Ethiopia reported as 22.9% (Hailemariam et al., 2012). Also, it is comparable with the psychological health problems of other frontline fitters, such as healthcare professionals, during the COVID-19 pandemic (Tsehay et al., 2020).

These study findings indicate that severe psychological health problems occur among police officers during the pandemic and due attention was not given for the importance of preventing and treating psychological health problems during the COVID-19 outbreak.

In the current study, some demographic factors influence psychological health problems during the COVID-19 pandemic. Age, female gender, having a chronic medical illness, low resilient coping score, and low social support were identified as risk factors for poor psychological health problems, such as depression, anxiety, and insomnia, as reported by previous studies. Being married is found to be a risk factor against previous literature because of the concern about being a source for infection of COVID-19 for loved ones and youngsters.

### Strengths

This study examined the prevalence and factors related to psychological health problems (i.e., symptoms of depression, anxiety, and insomnia) during the COVID-19 pandemic in Ethiopia among police officers. Our findings will serve as baseline information for policymaking, recognition of high-risk populations, and framework design for psychological crisis management of police officers.

## Conclusion and Recommendations

The rates of health symptoms, such as depression, anxiety, and insomnia, were found to be higher during the COVID-19 pandemic, especially among women, advanced age, married (has family), with chronic medical illness, low coping, and low social support. Psychological health interventions are urgently needed to fulfill demand during this outbreak. The police authorities and health sectors should work on strengthening individual coping status, and the concern should be given as other frontline fitters of the pandemic.

Future studies are needed to explore the association of the COVID-19 pandemic with psychological health problems in other parts of Ethiopia and in other countries and their long-term outcomes.

## Data Availability Statement

The raw data supporting the conclusions of this article will be made available by the authors, without undue reservation.

## Ethics Statement

The studies involving human participants were reviewed and approved by Institutional Review Board (IRB) from Wollo University College of medicine and health science. The patients/participants provided their written informed consent to participate in this study.

## Author Contributions

MT conceptualized and wrote the manuscript. MT, MN, HG, AB, and MB have contributed to the design, data collection tool preparation, writing proposal, and editing. All authors read and accepted the final manuscript.

## Conflict of Interest

The authors declare that the research was conducted in the absence of any commercial or financial relationships that could be construed as a potential conflict of interest.

## Publisher’s Note

All claims expressed in this article are solely those of the authors and do not necessarily represent those of their affiliated organizations, or those of the publisher, the editors and the reviewers. Any product that may be evaluated in this article, or claim that may be made by its manufacturer, is not guaranteed or endorsed by the publisher.
